# Stability and Repeatability Analysis of a Phase-Modulated Optical Fibre Sensor for Transformer Oil Ageing Detection

**DOI:** 10.3390/s25226851

**Published:** 2025-11-09

**Authors:** Ugochukwu Elele, Youssouf Brahami, Issouf Fofana, Azam Nekahi, Arshad Arshad, Kate McAulay

**Affiliations:** 1 School of Science and Engineering, Glasgow Caledonian University, Glasgow G4 OBA, UK; 2 Department of Applied Sciences, Université du Québec à Chicoutimi, Saguenay, QC G7H 2B1, Canada; ybrahami1@etu.uqac.ca (Y.B.);

**Keywords:** ageing, fiber optic sensor, transformer oil, refractive index, Fabry–Perot

## Abstract

Transformer oil ageing alters key physicochemical properties, notably the refractive index (RI), due to physical, particulate, and chemical changes. As a result, refractometric fibre-optic sensors have gained attention for enabling real-time monitoring and overcoming the limitations of traditional offline diagnostics. This study explores the use of a Fabry–Pérot phase-modulated fibre optic sensor (FISO FRI RI Sensor) for in-situ ageing detection in four industrial transformer oils: natural ester, synthetic ester, Nytro Bio 300X (vegetable-based), and Polaris GX (mineral-based). The oils were thermally aged under controlled conditions following degassing and drying. The sensor performance was evaluated using key metrics, including repeatability, thermal response, settling time, and linearity. Results show high repeatability (with standard deviations below 7 × 10^−5^ RIU and repeatability coefficients under 2 × 10^−4^ RIU), stable thermal response (~0.0004 RIU/°C), and strong thermal linearity (R^2^ > 0.99) across all samples. Natural ester and Nytro Bio 300X exhibited the most stable and consistent sensor responses, while synthetic ester and mineral oils showed greater variability due to temperature-induced RI shifts. These findings demonstrate the reliability and precision of this Fabry–Pérot phase-modulated sensor for online transformer oil condition monitoring, with strong potential for integration into smart grid diagnostics.

## 1. Introduction

Previous studies have highlighted in detail the importance of online ageing detection to circumvent the challenges of conventional offline ageing detection strategies, such as possible sample contamination during repeated sampling procedures, economic costs associated with the different diagnostic chains (sampling, transportation, diagnostics, analysis, and reporting), exposure to possible risks for personnel undertaking any element of the five diagnostic chains, person-hour loss, and poor adaptability for predictive maintenance practices [1,2,3,4].

Out of the various online ageing detection methods for transformer oil, such as cross capacitance sensors exploiting the changes in capacitance that follow dielectric property changes [5], horn antenna in an anechoic chamber exploiting the reflection coefficient from incident and reflected electromagnetic waves [6], photoacoustic spectroscopy (PAS) system exploiting the thermodynamic properties of dissolved gases [7]; optical fibre sensors stand out owing to their electromagnetic interference immunity, compact size, high sensitivity, extreme environment adaptability, and resistance to ionisation [3,8,9,10]. The key instrumentation variable for fibre-optic sensors is the refractive index, as transformer oil ageing markers such as moisture accumulation, darkening, particle size changes, acidity growth, and polar contaminant accumulation affect it.

Fibre optic sensors (particularly intensity-modulated optical fibre sensors) have reportedly been used for ageing detection studies in transformer oil. Using an optical spectrum analyser (OSA), the authors of [11] utilised a single-mode-multimode-single-mode (SMS) intensity modulation configuration to detect breakdown (BDV) values in transformer oil. The authors of [2] utilised a single-mode intensity modulation configuration for BDV marker identification in transformer oil. The authors of [10,12] utilised a U-shaped intensity modulation configuration for a sensitivity analysis study on various sucrose and transformer oils. Aside from the intensity modulation configuration, the wavelength modulation configuration has been previously used for ageing marker detection studies in transformer oil. The authors of [13,14] utilised the Fibre Bragg Grating (FBG) sensor, taking advantage of its wavelength modulation to track the BDV marker in transformer oil. Phase modulation optical fibre sensors, such as Fabry–Pérot interferometric sensors, are also utilised in transformer oil ageing monitoring, though their applications are less frequently reported than those of intensity-modulated optical fibre sensors. A more detailed literature review of various transformer oil online sensors conducted by the authors is available in [1]. Comparatively, the patented FISO FRI architecture used here combines white-light Fabry–Pérot sensing with Fizeau cross-correlation to return an absolute optical path difference (central correlation peak), thereby eliminating fringe-order ambiguity, preserving a wide RI range (1.000–1.700), and delivering high repeatability with a linear thermal coefficient (~ –0.0004 RIU °C^−1^) across four oil chemistries (Section 4.1, Section 4.2, and Section 4.3). This unique configuration directly addresses practical limitations in other configurations: intensity-modulated probes (U-shaped/SMS) are amplitude-based and thus susceptible to turbidity/scattering, alignment and source fluctuations, and surface fouling [4,10], whereas the FRI’s phase readout with white-light cross-correlation is less sensitive to absolute intensity and provides a stable central-peak metric even when throughput varies; FBG (wavelength-modulated) schemes require spectral tracking and show cross-sensitivities to temperature/strain, while the FRI yields absolute optical path difference (OPD) with a simple, linear thermal coefficient that facilitates straightforward temperature compensation [15,16]; and monochromatic Fabry–Pérot sensors suffer fringe-order ambiguity and restricted dynamic range, which the FRI overcomes via its white-light correlation to provide an unambiguous measurement across a broad RI span [17]. Additionally, the FRI-NP package offers EMI/RFI immunity, 0–100 °C operation, and a 1000 psi proof rating for in-situ deployment, consistent with the settling, repeatability, and thermal-linearity results reported here (see Table 1).

**Figure 1:**
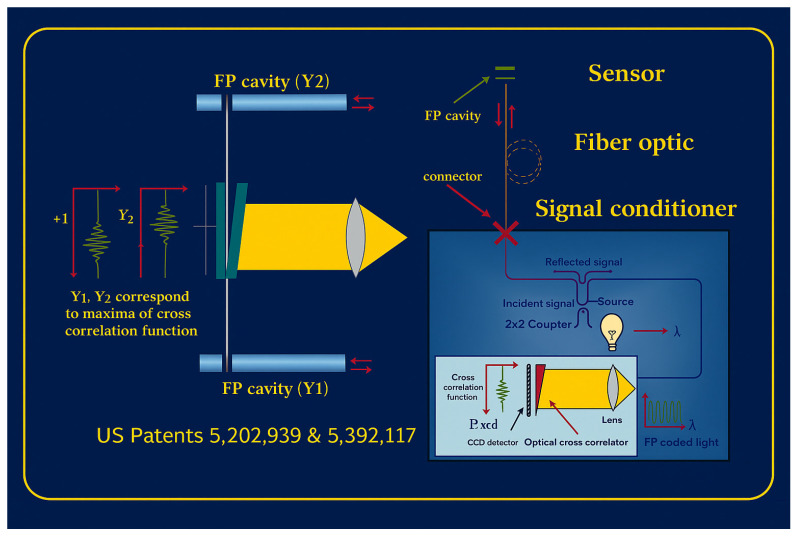
FISO FRI Sensor Diagram [22].

**Figure 2:**
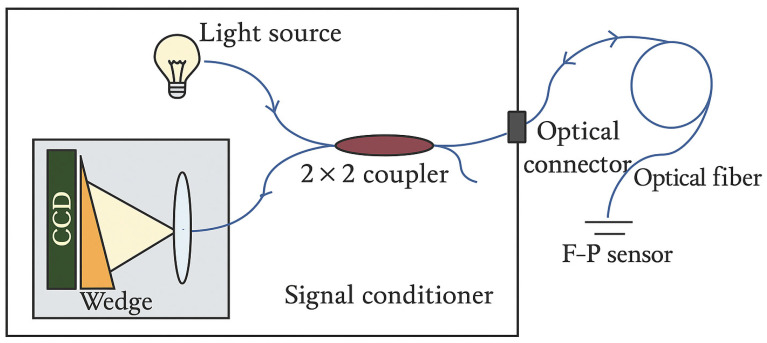
FISO FRI UMI Schematic Diagram [18].

In collaboration with FISO Resonetics, this study investigates the novel, patented FRI refractive index sensor (see Figure 1) for transformer oil ageing monitoring applications. The patented FISO FRI sensor employs a white-light Fabry–Pérot (F–P) interferometric principle that fundamentally differs from conventional fibre-optic refractive index measurement techniques, including traditional F–P approaches (see Table 1) [18,19]. Unlike typical F–P sensors, which often rely on monochromatic light and relative fringe counting, this sensor uses an incoherent broadband (white-light) source injected into a fibre-based F–P cavity containing the sample [18,19]. The emerging interference signal is reinjected into the fibre and processed using a Fizeau wedge cross-correlation scheme (see Figure 1 and Figure 2), producing a distinct central correlation peak corresponding to the optical path difference of the cavity. This configuration eliminates the fringe-order ambiguity and the limited dynamic range commonly associated with conventional F–P sensors, enabling highly precise, linear measurements of refractive index across a wide range. By reinjecting the interference signal into the fibre and using a robust cross-correlation readout, the system achieves enhanced stability, reproducibility, and suitability for in-situ applications, attributes not typically attainable with classical F–P or evanescent-field fibre-optic sensors [18,19]. This article presents an initial exploration of its use for industrial transformer oil applications, beginning with characterisation studies on four transformer oil samples. These studies focus on evaluating stability, repeatability, and temperature response, providing foundational insight into the sensor’s suitability for monitoring industrial oil systems.

## 2. Principle of Instrumentation

According to [19,20,21], a Fabry–Pérot cavity is designed by separating two parallel partially reflecting surfaces by a distance $d_{f}$, with a medium between the two surfaces having a refractive index, *n*. To enhance the functionality of the instrumentation, a partially reflective surface is employed to enable directed external light to enter the system (via the Universal Multichannel Instrument [UMI] and a multimode fibre) and exit the cavity (through the multimode fibre back to the UMI). This setup generates a spectrally varying transmission or reflection function due to the interference of multiple reflected waves. The integrated white-light source operates over 650–1000 nm with an effective central wavelength near 800 nm (see Table 2). This NIR window is appropriate for transformer oils, which are essentially transparent in this band, supporting low-loss interferometric measurements. Given the short optical path and low source power, illumination-induced self-heating is negligible. Constructive interference occurs when the reflected waves are in phase, producing a transmission peak, whereas destructive interference arises when the waves are out of phase, leading to diminished transmission [19,20].

The transmission function, $T_{f}$, is represented mathematically as [20,21]:

(1) $$T_{f}\left(n,\;\lambda ,\;d_{f}\right)\;=\;\frac{1}{\left(1+F\sin^{2}\left[\frac{2\pi nd_{f}}{\lambda }\right]\right)}$$

where $d_{f}$ represents the distance between the reflecting surfaces, λ represents the wavelength of the light, n represents the refractive index of the material between the cavity, F represents the finesse (the measure of the sharpness of transmission or reflection), which is a function of the mirror reflectance, R. Mathematically, the finesse is expressed as:

(2) $$F\left(R\right)\;=\;{\left[\frac{4R}{\left(1-R\right)}\right]}^{2}$$

The transmission function, as shown in Equation (1), explains that the Fabry–Pérot sensor can effectively be used for refractive index measurement, n, by keeping $d_{f},\;\lambda ,\;\mathrm{and}\;R$ constant [20,21].

According to Pinet [19], the FISO F-P sensor for refractive index (RI) measurement consists of a fixed cavity length (ensuring constant $d_{f}$), and the cavity is opened instead of being sealed, to allow non-viscous fluid passage whose RI value (typically between 1.0000 and 1.7000) is to be inferred. The FSIO Fabry–Pérot sensor specification is summarised in Table 2 below. The FISO FRI sensor employs a multimode optical fibre with a 50 µm core (125 µm cladding, per the IEC 60793 multimode specification), corresponding to the F1 configuration for the UMI system. The use of this fibre geometry increases broadband coupling efficiency and improves the optical throughput of the interferometric signal. Although multimode propagation can introduce modal dispersion, the very short Fabry–Pérot cavity length and the Fizeau cross-correlation readout scheme effectively suppress these effects, ensuring highly stable and repeatable refractive-index measurements.

## 3. Materials and Methods

### 3.1. Sample Preparation and Standards

**Figure 3:**
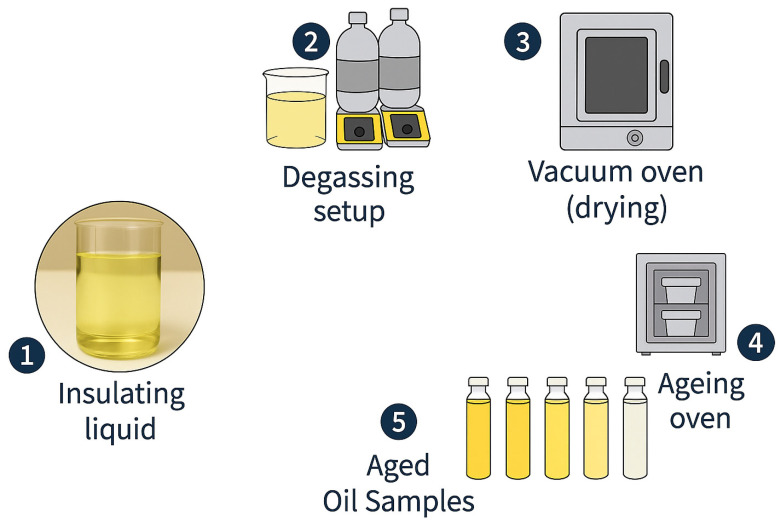
Sample Processing Steps.

For this study, primary experimental data were utilised. Fresh industrial transformer oils comprising Polaris GX mineral oil, Midel eN 1204 natural ester oil, Midel eN 7131 synthetic ester oil, and Nytro Bio 300X vegetable oil were degassed and dried to remove residual moisture and dissolved gases such as oxygen, nitrogen, and hydrogen absorbed from varying environmental conditions, which can interfere with the ageing process. By employing degassing and drying processes, the ageing process is primarily influenced by experimental conditions, thereby enhancing the reproducibility of measurement data and providing a good baseline for effectively monitoring the effect of ageing on transformer oil. A Fisher Scientific Fisher Brand Maxima C Plus Vacuum Pump (Waltham, MA, USA), set to −1 Bar vacuum pressure, and a magnetic stirrer, ensuring a uniform degassing process, were used (see Figure 3 below). The degassing process lasted 48 h. A Fisher Scientific Isotemp Vacuum Oven, Model 285A (Waltham, MA, USA), set at −1 Bar Vacuum pressure and 60 °C non-ageing drying temperature, was used for the drying process for 48 h (see Figure 3 below). Table 3 summarises the transformer oil specifications and compliance with relevant standards.

The Procedure B ageing method was utilised in this work, as per the ASTM D1934-20 [23] standard. Metal catalysts (copper, 3 g/L; 3 cm × 3 cm surface area) were used to increase the oxidation rate. Ageing was performed at an accelerated temperature of 115 °C (as per ASTM D1934-20) to produce representative laboratory-aged samples equivalent to field ageing. The 96-h sampling interval was strictly adhered to. A Fisherbrand oven (see Figure 3 above) capable of maintaining a constant temperature of 115 °C was used. Aluminium troughs with loose seals to allow minimal oxygen ingress were used. This setup emulates a free-breathing transformer as commonly found in the field and also promotes oxidative ageing. A total of 20 distinct samples (samples 0 to 4; oil + 3 g/L copper) for all four transformer oil types were obtained from this process.

### 3.2. Fibre Optic Sensor Setup and Calibration

**Figure 4:**
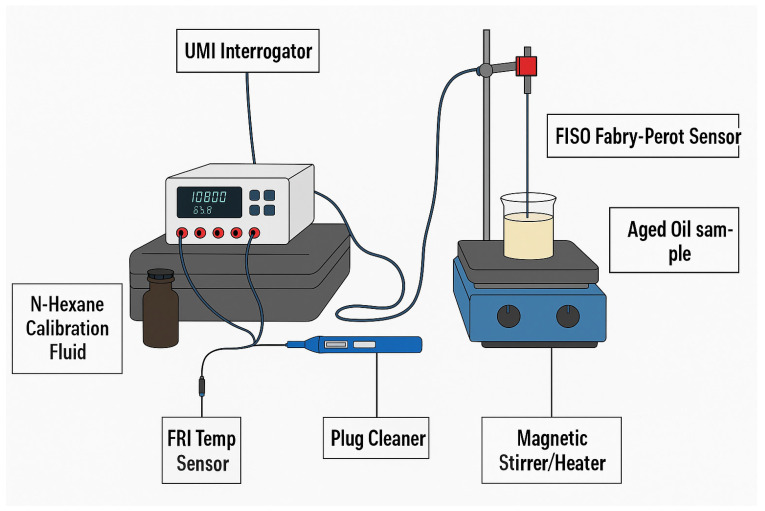
FISO Fabry–Pérot Instrumentation Setup.

The instrumentation system comprises a Fabry–Pérot interferometer sensor and a multimode fibre-optic cable, with a white-light source via the FISO Universal Multichannel Instrument (UMI), as shown in Figure 4 below. FISO UMI interfaces with the FISO Commander 2 software, which enables graphical visualisation, configuration, and data acquisition. The UMI also includes analogue output channels that enable real-time data acquisition via microcontrollers and legacy softwares such as MATLAB (R2021a) and LabVIEW (NXG 5.1). The sensor output is not sensitive to twists in the fibre optic cable.

**Figure 5:**
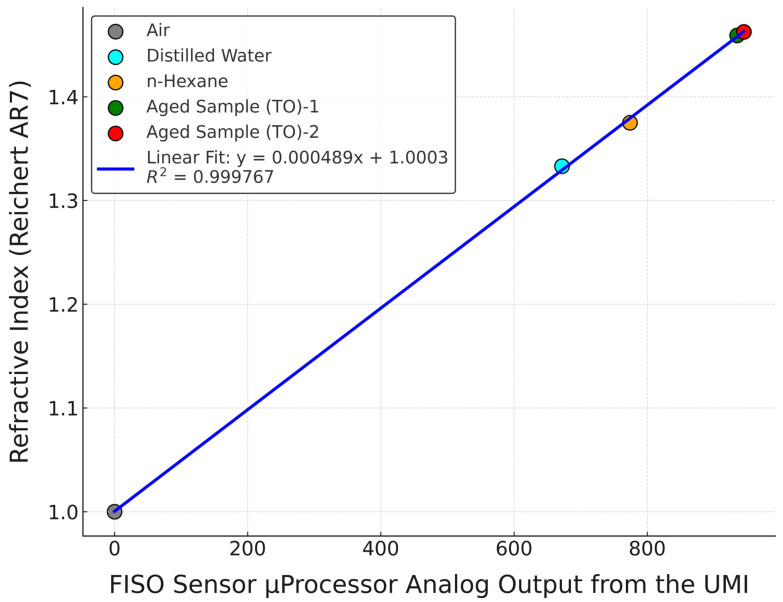
FISO Fabry–Pérot RI Calibration Plot.

The following steps were taken for measurement data acquisition:

1. The measurement unit was set up as shown in Figure 4, and the FISO Commander 2 software was initialised and confirmed to be communicating with the UMI.
2. The Fabry–Pérot sensor was calibrated correctly. When exposed to ambient air, the refractive index was approximately 1.0000. Alternatively, the Fabry–Pérot sensor was calibrated using samples of known refractive index (N-Hexane = 1.3749 RIU). The sensor was inserted into the beaker containing pure N-hexane and calibrated through the UMI. See Figure 5 and Table 4 for the calibration graph and table, respectively.
3. Once calibrated, the sensor was allowed to dry in the air to prevent sample contamination. A dried sensor reported a refractive index value of approximately 1.0000. A test sample of 40 mL was poured into a beaker, and a magnetic stirrer bar was added. The beaker was placed on a stirrer, and the sensor was inserted into the test sample.
4. Through the FISO Commander 2 software, the refractive index values and corresponding sample temperature were acquired and stored for further analysis.
5. After each measurement, the sensor probe was cleaned by inserting it (in a suspended position) into the *n*-hexane bottle. A properly cleaned probe read a refractive index value near 1.3749 RIU.
6. Steps 3 to 5 were repeated for the remaining test samples.

## 4. Results

### 4.1. Settling Time Analysis

**Figure 6:**
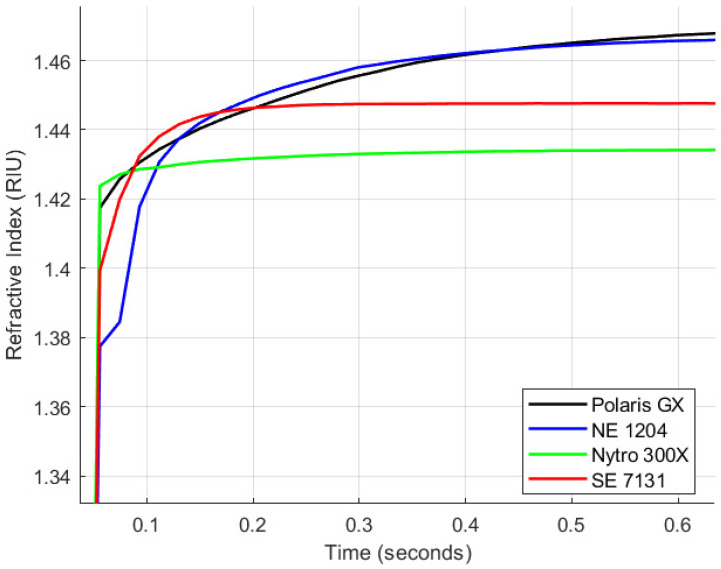
Settling Time Analysis.

The purpose of settling time studies is to evaluate how quickly the sensor stabilises following a change in the oil sample or environmental conditions. This analysis is particularly important for online monitoring applications, where transformer oil properties may change rapidly due to faults, contamination, or ageing. The experimental setup detailed in Section 3.2 was employed for this analysis. The calibrated sensor was immersed in a stirred 40 mL of zero-hour test samples of Polaris GX, NE 1204, SE 7131, and Nytro Bio 300X. Data acquisition commenced at the point of insertion using the FISO Commander 2 software. The results are presented in Figure 6 and Table 5. Data were collected over 2.24 min at a sampling interval of 1.20 s, under ambient conditions at 22 °C.

The results indicate that the sensor reaches over 99% of its steady-state value in under 0.5 min for all oil samples, demonstrating its high responsiveness to changes in operating conditions—an essential attribute for in-situ transformer monitoring. Among the tested samples, Synthetic Ester Oil SE 7131 exhibited the fastest response, reaching 100% of the steady-state value within 36 s. In contrast, Polaris GX mineral oil showed the slowest response, reaching 99.596% in the same timeframe, a level achieved earlier by the other oils. Overall, the sensor’s settling time performance is highly commendable, confirming its suitability for high-voltage transformer applications.

### 4.2. Repeatability Studies

**Figure 7:**
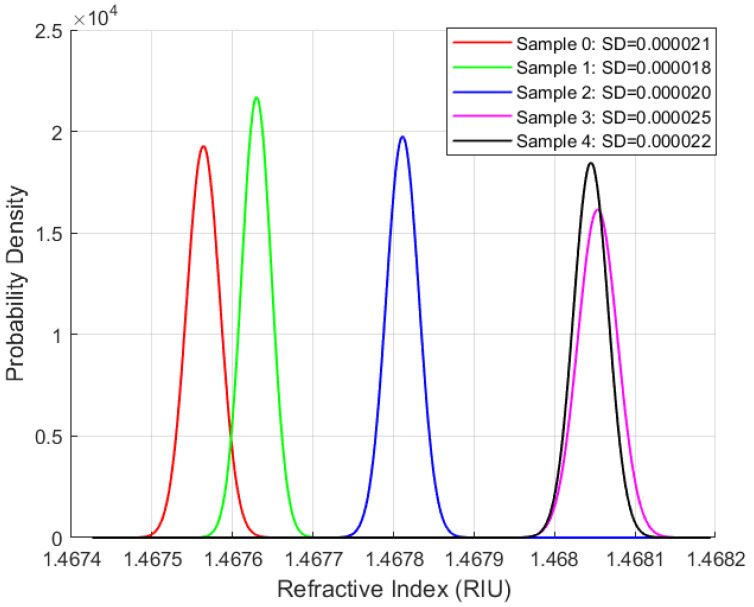
Repeatability Results for NE 1204 Samples.

**Figure 8:**
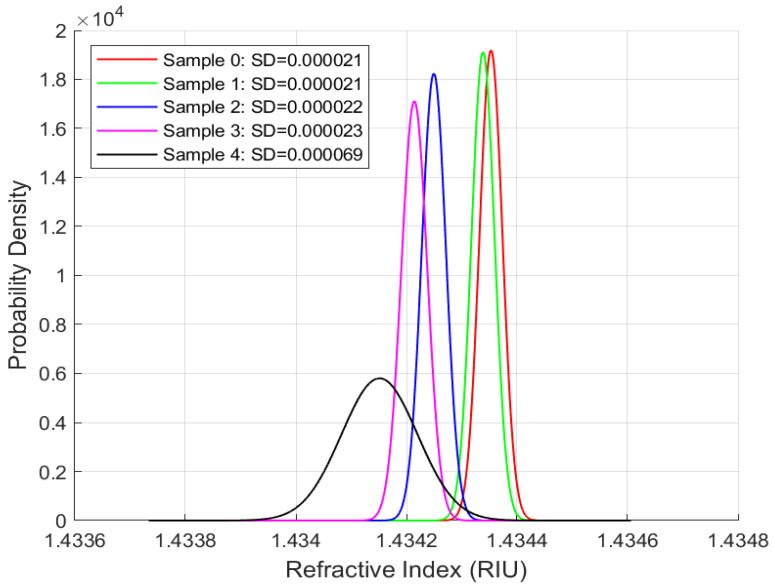
Repeatability Results for Nytro Bio 300X Samples.

**Figure 9:**
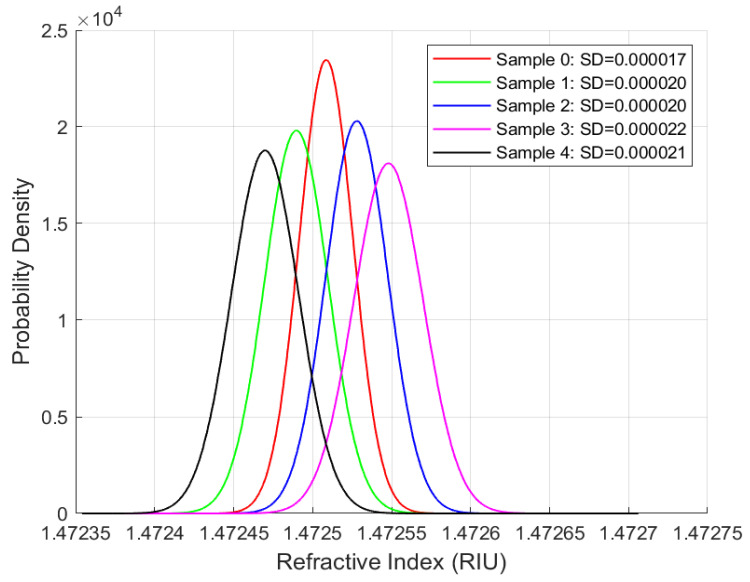
Repeatability Results for Polaris GX Samples.

**Figure 10:**
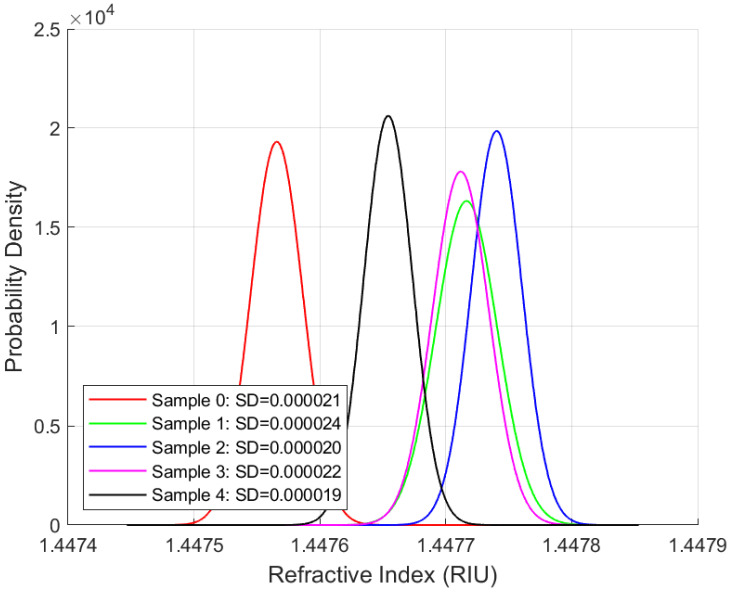
Repeatability Results for SE 7131 Samples.

Repeatability studies were performed for the phase-modulated fibre-optic sensor using all four oil samples at the same sampling rate and duration. The objective is to assess the sensor’s precision and its ability to deliver consistent measurements over time, a key requirement for long-term applications. The standard deviation is the primary metric for evaluating measurement variability. Figure 7, Figure 8, Figure 9 and Figure 10 present the distribution plots illustrating the standard deviation for each oil type under various ageing conditions at room temperature.

A consistent observation across Figure 7, Figure 8, Figure 9 and Figure 10 is that the FISO Fabry–Pérot fibre optic sensor demonstrated exceptionally high repeatability, with values averaging approximately 0.00002 RIU across all four oil types, irrespective of ageing condition. This high level of precision underscores the sensor’s suitability for high-voltage applications. Although the repeatability values shown in Figure 7, Figure 8, Figure 9 and Figure 10 are nearly uniform, further examination of the distribution plots reveals that the natural ester oil (NE 1204) and the Nytro Bio 300X samples exhibit the most distinct and interpretable trends. These two oils show minimal peak overlap and consistent directional patterns—left to right for NE 1204 and right to left for Nytro Bio 300X (owing to the presence of antioxidants in Nytro Bio 300X).

In contrast, the synthetic ester oil and mineral oil samples display less consistent trend behaviours. The clearer distribution patterns and reduced peak overlap observed in NE 1204 and Nytro Bio 300X suggest their greater suitability for instrumentation applications, as they enable more effective differentiation between ageing states. These findings are further supported by the thermal response analysis results discussed in the subsequent section.

According to Table 6, Table 7, Table 8 and Table 9 below and Figure 7, Figure 8, Figure 9 and Figure 10 above, the standard deviation (SD) of repeated readings under identical conditions remained within (2–7) × 10^−5^ RIU for all samples, corresponding to repeatability coefficients $R_{c}$ below 2 × 10^−4^ RIU. These results demonstrate excellent measurement stability of the Fabry–Pérot sensor.

Across all oil types, one-way ANOVA tests revealed statistically significant differences among ageing states (*p* < 0.001), confirming that the observed variations in mean ($\bar{x})$ refractive index are not due to random measurement noise.

The combination of low SD, narrow confidence intervals, $\left({CI}_{95}\right)$, very low standard error of the mean, $s_{\bar{x}},$ and highly significant ANOVA results confirm both the repeatability and sensitivity of the sensor system across oil chemistries.

### 4.3. Thermal Response Analysis

**Figure 11:**
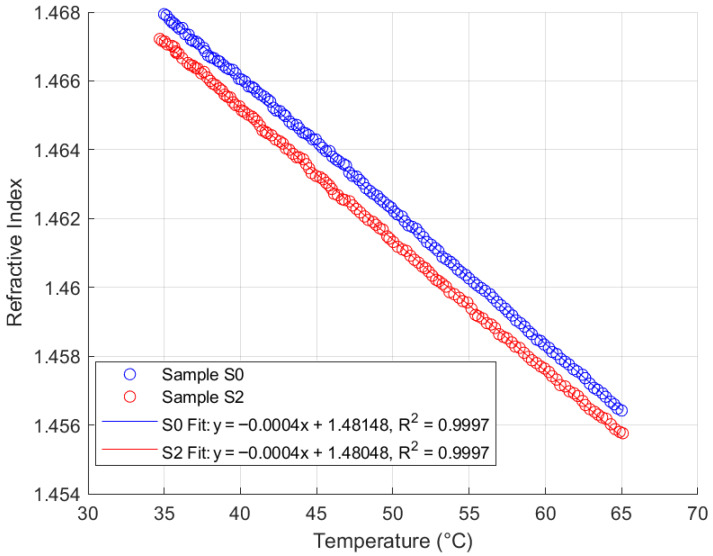
Fabry–Pérot FRI Thermal Response (Mineral Oil).

**Figure 12:**
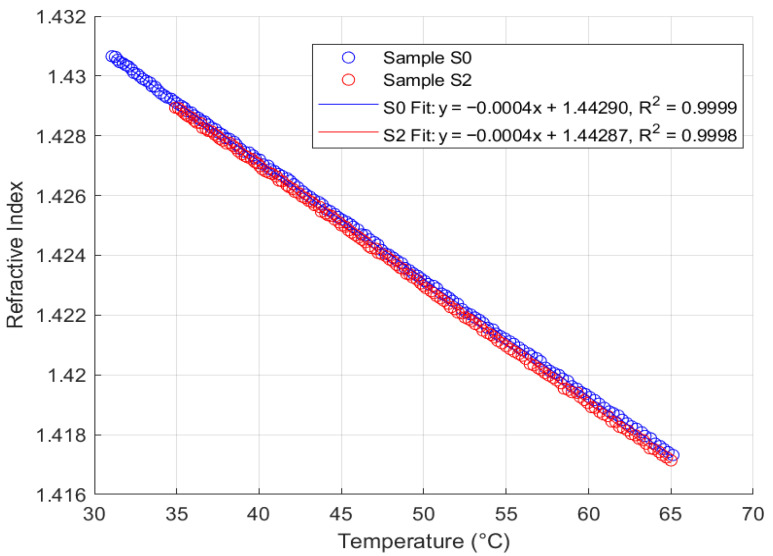
Fabry–Pérot FRI Thermal Response (Nytro Bio 300X).

**Figure 13:**
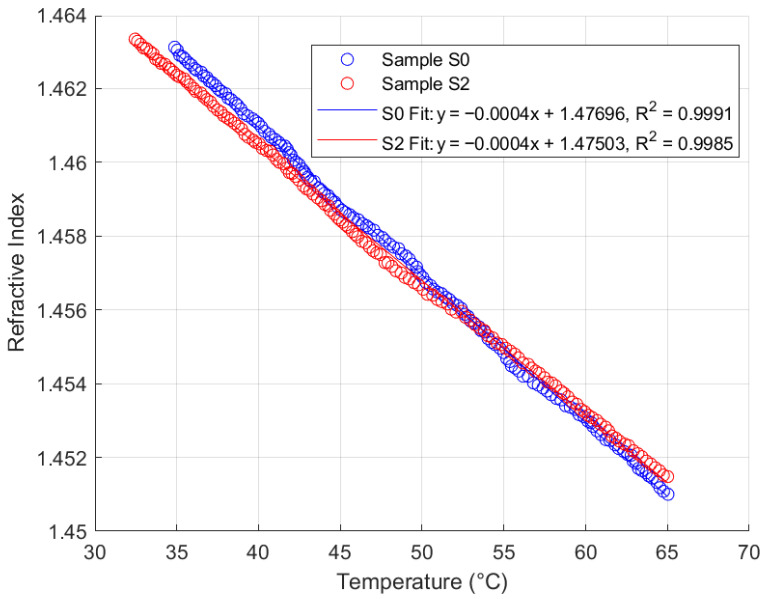
Fabry–Pérot FRI Thermal Response (NE 1204).

**Figure 14:**
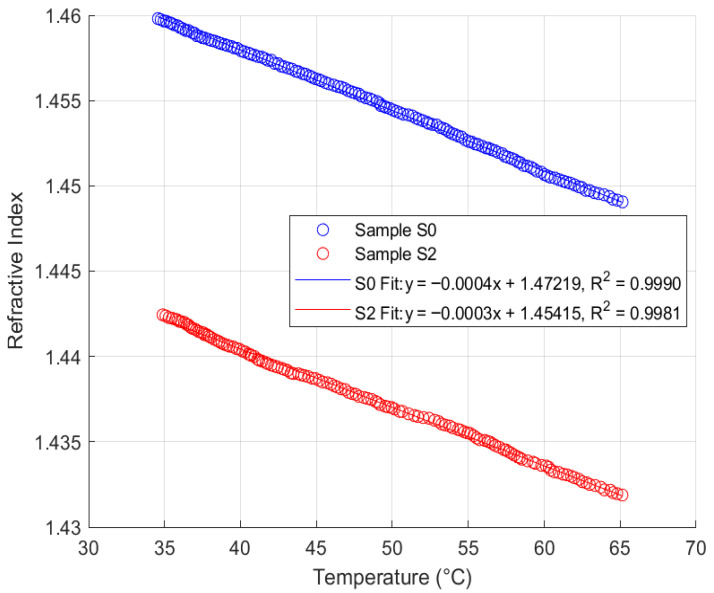
Fabry–Pérot FRI Thermal Response (SE 7131).

Thermal response analysis was performed on the FISO Fabry–Pérot FRI sensor with samples S0 and S2 from each of the four oil types, across a temperature range of 35 °C to 65 °C, maintaining a similar sampling rate. This range reflects typical operating temperatures for in situ transformers. The sensor responses for samples S0 and S2 are summarised in Figure 11, Figure 12, Figure 13 and Figure 14.

Analysis of Figure 11, Figure 12, Figure 13 and Figure 14 reveals that the FISO Fabry–Pérot FRI sensor exhibits a negative linear thermal response across all samples, regardless of ageing condition. This linearity facilitates straightforward compensation for temperature variations during in situ transformer oil measurements. Alternatively, a temperature control mechanism could be integrated into the measurement system to either maintain the sampled oil at the calibration temperature or ensure that refractive index readings are taken only at the calibration temperature. The sensor’s thermal coefficient, α, is consistently measured at −0.0004 RIU/°C, corresponding to the slope of the fitted regression lines. The intercepts represent the extrapolated refractive indices at 0 °C for each sample.

Consistent with the repeatability analysis presented in Section 4.2, the natural ester oil (NE 1204) and Nytro Bio 300X exhibited the most consistent thermal response, with minimal deviation even between samples aged 192 h apart. This indicates greater resistance to temperature-induced measurement variation than the other two oil types. Additionally, these oils enable more straightforward temperature compensation using a linear model, as their thermal coefficient (α) remains constant and the intercepts for aged samples converge closely to similar values. These findings support the observations discussed in Section 2.

## 5. Discussion, Conclusions, and Future Works

Refractometric fibre-optic sensors play a pivotal role in online detection of ageing in transformer oils by leveraging refractive index (RI) variations that occur as the oil degrades over time. This experimental study demonstrates that the patented FISO Fabry–Pérot phase-modulated fibre-optic sensor has strong potential for monitoring transformer oil ageing, owing to its precision, fast settling time, and resilience under varying operating conditions.

In terms of key instrumentation performance metrics—particularly repeatability—the Fabry–Pérot interferometric refractometric fibre-optic (FRI) sensor consistently delivered low standard deviations and repeatability coefficient across different transformer oil types and stages of thermal ageing. In addition, one-way ANOVA tests indicated statistically significant differences among ageing states for all oils (*p* < 0.001), demonstrating that the observed variations are not due to random measurement noise. This high repeatability, combined with the sensor’s inherent immunity to external disturbances such as mechanical twisting, positions it as a robust and reliable alternative to conventional intensity-modulated fibre optic sensors for continuous, real-time ageing diagnostics.

The study further revealed that, based on repeatability and thermal response analyses, natural ester oil and Nytro Bio 300X (a vegetable-based oil) yielded superior performance outcomes compared to synthetic ester oil and Polaris GX mineral oil. Specifically, the Fabry–Pérot sensor maintained a consistent thermal coefficient of approximately 0.0004 RIU/°C, with fitted linear plots exhibiting coefficients of determination exceeding 99% across all oil types. In contrast, the synthetic ester and mineral oils demonstrated greater sensitivity to temperature-induced RI changes, leading to increased measurement variability and lower consistency in thermal analysis plots.

Additionally, the Fabry–Pérot FRI sensor exhibited fast settling times for both fresh and aged samples across all four oil types, highlighting its ability to provide consistent and timely responses under various ageing conditions. These findings reinforce the sensor’s effectiveness and reliability as a precision diagnostic tool for transformer oil ageing monitoring applications.

The FISO FRI refractive index sensor used in this work is a patented commercial device, and as such, some internal calibration and signal-processing parameters are not disclosed by the manufacturer (FISO–Resonetics) to end users. These include, for example, the detailed internal correlation algorithm used in the Fizeau wedge signal-processing module and specific optical cavity parameters embedded in the proprietary firmware of the Universal Multichannel Instrument (UMI). While these parameters are inaccessible, the authors have ensured that all experimentally measurable quantities, such as the refractive index range, resolution, operating wavelength, and temperature range, are reported (Table 2 of the manuscript). In addition, the authors have provided a comprehensive operational description of the measurement principle and calibration procedure in Section 3.2 to ensure experimental transparency and reproducibility.

Compared with intensity-based probes, which are affected by turbidity and alignment, and wavelength-based systems, which require spectral tracking and temperature or strain correction, the white-light, phase-based FRI sensor provides a direct, unambiguous measurement of optical path difference. It can measure refractive indices from 1.000 to 1.700, achieves over 99% stability in less than half a minute under the reported test conditions, and uses fibre-optic, EMI-resistant, pressure-rated hardware suitable for in-situ applications.

Despite high short-term repeatability and linear thermal behaviour reported, several practical limits remain. (1) The readout is temperature-dependent; field use therefore requires temperature control or linear compensation. (2) Optical throughput may decline in harsh service due to microfouling/varnish or increased scattering; this mainly lowers fringe contrast rather than biasing phase, but can reduce margin in heavily degraded oils and thus warrants periodic probe cleaning/inspection. (3) A multi-month drift study was not performed; long-term stability will depend on source stability, connector integrity, and fouling rates, and is the subject of planned field trials. (4) Certain internal parameters of the commercial interrogator are proprietary; to support reproducibility, all measurable, user-level specifications (operating band 650–1000 nm, RI range 1.000–1.700, operating temperature 0–100 °C, 1000 psi proof pressure for FRI-NP) and the procedures used here are reported.

Future research would explore advanced temperature-compensation models tailored to the linear temperature response of this sensor, optimised for different oil types and operational temperature ranges. This would enhance the reliability of ageing diagnostics by minimising the impact of temperature-induced RI fluctuations. Furthermore, the potential benefits of sample dilution in improving sensor accuracy and stability merit further investigation, particularly for highly viscous or degraded oil samples. Additional comparative studies between the FISO FRI sensor and other commercial sensors can also be conducted, especially in precision, stability, and thermal-response tests.

## Figures and Tables

**Table 1:** Comparison Table (FISO FRI vs. Conventional Sensors).

Feature	Conventional F-P RI Sensors	Evanescent/Tapered Fibre RI Sensors	FISO FRI Sensor (Patented)
Light Source.	Monochromatic (single wavelength).	Typically monochromatic.	Broadband (white-light).
Measurement Type.	Relative fringe shifts.	Surface/Evanescent field interaction.	Absolute optical path difference via cross-correlation.
Fringe Ambiguity.	Yes, fringe order can be ambiguous.	n/a.	No, the central correlation peak gives an unambiguous reading.
Dynamic Range.	Limited.	Moderate.	Wide range (1.000–1.700 RI).
Signal Processing.	Direct fringe counting.	Minimal.	Fizeau wedge cross-correlation readout.
Industrial Applicability.	Limited.	Limited.	High (in-line monitoring, harsh conditions).
Operating Wavelength Band.	Narrow line; depends on source (e.g., 633/850/1310 nm).	Narrow line; design-dependent.	650–1000 nm (nominal ~800 nm).
RI range.	Typically narrow, design-dependent.	Moderate; depends on coating/taper.	1.000–1.700 RI.
Operating Temperature.	Design-dependent.	Design-dependent.	0–100 °C (sensor).
Proof pressure/packaging.	Lab-scale optics; limited.	Lab-scale; limited pressure.	FRI-NP proof 1000 psi; fibre minimum bend radius 2.5 cm.

**Table 2:** FISO Fabry–Perot Sensor Specification [18].

S/*n*	Parameter	Value
1.	Refractive Index Range.	1.000 to 1.700 RI.
2.	Resolution.	0.0001 RI.
3.	Operating Temperature.	0 °C to 100 °C.
4.	Storage Temperature.	−30 °C to 80 °C.
5.	Minimum Bend Radius.	2.5 cm.
6.	Proof Pressure of the FRI-NP Model.	1000 psi (max).
7.	Measurement Wavelength.	650 nm to 1000 nm (800 nm preferable; NIR Range).
8.	Fibre type/geometry.	Multimode optical fibre, 50 µm core/125 µm cladding (F1 configuration for UMI; standard per IEC 60793).

**Table 3:** Transformer Oil Specification and Compliance.

S/N	Parameters	Midel eN 1204	Midel eN 7131	Polaris GX	Nytro Bio 300X
1.	Source.	Rapeseed/Canola Oil.	Synthetic.	Crude Petroleum.	Non-Specified Plant Sources.
2.	Biodegradable.	Yes.	Yes.	No.	Yes.
3.	Dielectric Strength compliance: ≥35 kV as per BS EN 62770:2014.	Yes.	Yes.	Yes.	Yes.
4.	Moisture Content Compliance: <200 mg/kg as per BS EN 62770:2014.	Yes.	Yes.	Yes.	Yes.
5.	Acidity Compliance: <0.06 mgKOH/g as per BS EN 62770:2014.	Yes.	Yes.	Yes.	Yes.
6.	Fire Point Compliance: ≥300 °C as per BS EN 62770:2014.	Yes.	Yes.	Yes.	Yes.

**Table 4:** Calibration data for the FISO FRI refractive index sensor.

Material	FISO Sensor µ_Processor Analogue Output from the UMI	Refractive Index (at 20 °C, 589 nm) Using Reichert AR7 Refractometer
Air	0	1.0000
Distilled water	672	1.3330
N-Hexane	774	1.3749
Random Aged Sample (TO)	935	1.4591
Random Aged Sample (TO)	945	1.4625

**Table 5:** Settling Time Analysis Summary Data.

Oil	State	t_10_–_90_ (s)	t_95_ (s)	t_99_ (s)	%SS @ 36 s
SE 7131 (synthetic ester)	S0	<12	≤12	≤12	100.00
Polaris GX (mineral)	S0	<12	≤12	≤24	99.596
NE 1204 (natural ester)	S0	<12	≤12	≤24	99.843
Nytro Bio 300X (vegetable)	S0	<12	≤12	≤12	99.983
Conditions:	ambient 22 °C; 40 mL; 1.20 s sampling; stirring: Yes				
Definitions:	t_10_–_90_: rise time from 10%→90% of the step; t_95_/t_99_: first timestamps where the normalised response ≥ 95%/≥ 99% of steady state; %SS @ 36 s: percentage of steady state reached at 36 s.				

**Table 6:** Repeatability Summary Data for Nytro Bio 300X Oil.

Nytro Bio 300X Oil; Anova *p*_Value < 0.001							
Age	*n*	$\bar{x}$ [RIU]	SD [RIU]	$R_{c}$ [RIU]	$C_{v}$	$s_{\bar{x}}$ [RIU]	${CI}_{95}$ [RIU]
S0	99	1.434353	0.000021	0.000059	0.000015	0.000002	0.000004
S1	99	1.434338	0.000021	0.000058	0.000014	0.000002	0.000004
S2	99	1.434251	0.000022	0.000061	0.000015	0.000002	0.000004
S3	99	1.434216	0.000022	0.000062	0.000016	0.000002	0.000004
S4	99	1.434150	0.000070	0.000195	0.000049	0.000007	0.000014

**Table 7:** Repeatability Summary Data for Polaris GX Oil.

Polaris GX Mineral Oil; Anova *p*_Value < 0.001							
Age	*n*	$\bar{x}$ [RIU]	SD [RIU]	$R_{c}$ [RIU]	$C_{v}$	$s_{\bar{x}}$ [RIU]	${CI}_{95}$ [RIU]
S0	99	1.472508	$2\times 10^{-5}$	$5\times 10^{-5}$	$1\times 10^{-5}$	$2\times 10^{-6}$	$3\times 10^{-6}$
S1	99	1.472490	$2\times 10^{-5}$	$5\times 10^{-5}$	$1\times 10^{-5}$	$2\times 10^{-6}$	$4\times 10^{-6}$
S2	99	1.472528	$2\times 10^{-5}$	$6\times 10^{-5}$	$1\times 10^{-5}$	$2\times 10^{-6}$	$4\times 10^{-6}$
S3	99	1.472548	$2\times 10^{-5}$	$6\times 10^{-5}$	$2\times 10^{-5}$	$2\times 10^{-6}$	$4\times 10^{-6}$
S4	99	1.472470	$2\times 10^{-5}$	$6\times 10^{-5}$	$2\times 10^{-5}$	$2\times 10^{-6}$	$4\times 10^{-6}$

**Table 8:** Repeatability Summary Data for NE 1204 Oil.

NE 1204 Natural Ester Oil; *p*_Value < 0.001							
Age	*n*	$\bar{x}$ [RIU]	SD [RIU]	$R_{c}$ [RIU]	$C_{v}$	$s_{\bar{x}}$ [RIU]	${CI}_{95}$ [RIU]
S0	99	1.467565	$2\times 10^{-5}$	$5\times 10^{-5}$	$1\times 10^{-5}$	$2\times 10^{-6}$	$4\times 10^{-6}$
S1	99	1.467630	$2\times 10^{-5}$	$5\times 10^{-5}$	$1\times 10^{-5}$	$2\times 10^{-6}$	$4\times 10^{-6}$
S2	99	1.467812	$2\times 10^{-5}$	$6\times 10^{-5}$	$1\times 10^{-5}$	$2\times 10^{-6}$	$4\times 10^{-6}$
S3	99	1.468055	$3\times 10^{-5}$	$7\times 10^{-5}$	$2\times 10^{-5}$	$2\times 10^{-6}$	$5\times 10^{-6}$
S4	99	1.468046	$2\times 10^{-5}$	$6\times 10^{-5}$	$1\times 10^{-5}$	$2\times 10^{-6}$	$4\times 10^{-6}$

**Table 9:** Repeatability Summary Data for SE 7131 Oil.

SE 7131 Synthetic Ester Oil; Anova *p*_Value < 0.001							
Age	*n*	$\bar{x}$ [RIU]	SD [RIU]	$R_{c}$ [RIU]	$C_{v}$	$s_{\bar{x}}$ [RIU]	${CI}_{95}$ [RIU]
S0	99	1.447567	$2\times 10^{-5}$	$6\times 10^{-5}$	$1\times 10^{-5}$	$2\times 10^{-6}$	$4\times 10^{-6}$
S1	99	1.447718	$2\times 10^{-5}$	$7\times 10^{-5}$	$2\times 10^{-5}$	$2\times 10^{-6}$	$5\times 10^{-6}$
S2	99	1.447742	$2\times 10^{-5}$	$5\times 10^{-5}$	$1\times 10^{-5}$	$2\times 10^{-6}$	$4\times 10^{-6}$
S3	99	1.447714	$2\times 10^{-5}$	$6\times 10^{-5}$	$2\times 10^{-5}$	$2\times 10^{-6}$	$4\times 10^{-6}$
S4	99	1.447655	$2\times 10^{-5}$	$5\times 10^{-5}$	$1\times 10^{-5}$	$2\times 10^{-6}$	$4\times 10^{-6}$

## Data Availability

The original contributions presented in this study are included in the article. Further inquiries can be directed to the corresponding author.
